# Development and validation of two analytical strategies for the determination of glucosides of acidic herbicides in cereals and oilseed matrices

**DOI:** 10.1007/s00216-023-04898-y

**Published:** 2023-08-14

**Authors:** Ivan Aloisi, Hans Mol

**Affiliations:** Wageningen Food Safety Research (WFSR), Part of Wageningen University & Research, Wageningen, the Netherlands

**Keywords:** Pesticides, Glucoside metabolites, Acidic herbicides, Deconjugation, Hydrolysis, Baby food

## Abstract

The aim of the present research was the development and validation of a selective and reliable method for the indirect and direct determination of acidic herbicide glucosides. Enzymatic deconjugation was investigated as a mild alternative to harsh alkaline hydrolysis. Various enzymatic options for deconjugation were exploited. One out of nine tested specific enzymes proved to be practical and repeatable for different matrices and concentration ranges, leading to the complete deconjugation of the glucosides. The method was validated according to the SANTE/11312/2021 guideline for cereals and oilseeds and for a rice-based infant formula. Additionally, for four acidic herbicide glucosides available on the market, a quantitative method for direct determination of the intact glucosides was optimized and validated. In both methods, the average recoveries were within 70–120%. The limits of quantification (LOQ) achieved were 10 µg kg^−1^ and 2.5 µg kg^−1^ for the intact glucosides and the free acids in cereal and oilseeds. For the rice-based infant formula, the LOQ was 1 µg kg^−1^ (3 µg kg^−1^ for dichlorprop). To confirm its applicability, the deconjugation approach was tested for fifteen samples (cereals, oilseeds, and citrus) with incurred residues. Comparisons were made between the method without deconjugation, and two methods with deconjugation, the here proposed enzymatic deconjugation and the more commonly used alkaline hydrolysis. The inclusion of enzymatic deconjugation during sample preparation led to an increase up to 2.7-fold compared to analysis without deconjugation. Enzymatic deconjugation resulted in comparable results to alkaline hydrolysis for 13 out of 15 samples.

**Graphical Abstract:**

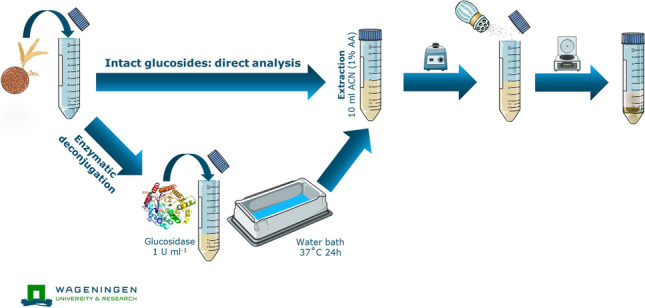

**Supplementary information:**

The online version contains supplementary material available at 10.1007/s00216-023-04898-y.

## Introduction

Pesticides can be absorbed by the plants, becoming a target of primary or secondary metabolism depending on the molecular structures. The molecular size determines its phyto-availability and also the main diffusion process [1]. Phenoxy acidic herbicides such as 2,4-dichlorophenoxyacetic acid (2,4-D), 2-methyl-4-chlorophenoxyacetic acid (MCPA), dichlorprop, and haloxyfop can be applied as free acid, salt, or ester. Esters (more lipophilic) tend to better penetrate the leaves, after which they are hydrolyzed into the free acids responsible for the herbicidal activity.

After being absorbed and distributed, there are three main metabolic phases through which a pesticide (or any other xenobiotic) can be bio transformed in plants, and within these phases, several are the enzymes and co-factors that play a crucial role [2]. Glucosylation of xenobiotics is one of the main phase II metabolism routes, and it plays an essential function as a defense mechanism. This process occurs by the addition of a sugar molecule (primarily glucose) to the native compound or to the phase I metabolite if the primary compound does not intrinsically contain a functional group in its chemical structure. Other possible types of conjugation take place with the addition of amino acids, fatty acids, and alcohol molecules. Nevertheless, the main phase II metabolic pathway in plants results on the generation of the respective glucoside metabolite.

Although the conjugates are not easily absorbed by the animals or humans (due to their high polarity), it can be assumed that the chance to be hydrolyzed in the gastrointestinal tract, thus releasing the native active compounds, is quite high. For this reason, during the last decade, the residue definitions (RDs) and the maximum residue levels (MRLs) of many pesticides were re-evaluated in accordance with Article 12 of Regulation 396/2005/EC [3], and in a number of cases include conjugates and esters besides the parent compound. The majority of pesticides with a carboxy- or phenolic substituent include conjugates in the RD [4]. Inclusion of the glucoside metabolites as such is only possible for a limited number of compounds for which the analytical reference standards are available. In other cases, only indirect determination is possible after deconjugation and determination of total free acid content. For this purpose, the most common procedure is based on the use of strong acidic and/or alkaline conditions to release the sugar from the parent pesticide. However, in some cases, this approach can generate a further degradation of the molecule, leading to the inability to correctly quantify the parent compound. The use of milder conditions, such as enzymatic deconjugation, can avoid this. Despite the fact that esters are relevant from a regulation point of view, there are two main reasons why they are often not included in existing methods. Firstly, the number of esters per single pesticide can be very high. For instance, there are 23 different esters of the herbicide 2,4-D commercially available. Thus, the development of a method capable of detecting all the different esters of several pesticides results in a very complex task. Additionally, after the application in the field, esters are rapidly hydrolyzed to the free and active form, reason why their content into the final product is usually negligible. The rapid conversion of esters into the free acidic forms and the importance of monitoring the glucoside conjugates are detailed described by the WHO/FAO Joint Meeting on Pesticide Residue (JMPR) reports for 2,4-D, MCPA, and haloxyfop [5–7]. Although there is no report available for dichlorprop, the same conclusion can be applied based on the chemical structure it has in common with 2,4-D and MCPA. These reports not only confirm the low stability of the esters as such, but also emphasize the need for methods capable of detecting conjugates.

In the past, several studies have been published, in which the deconjugation step was tested for the evaluation of the total content of acidic pesticides in cereal-based commodities. Most of these investigations described a comparison of extraction techniques with and without the deconjugation process.

One of the first studies was reported in 1971 by Chow et al. [8]. The investigation was focused on the determination of the presence of conjugates of MCPA in treated wheat, and on the evaluation of alkaline hydrolysis (before the addition of extraction solvent) for the quantification of the total herbicide residue. The authors reported an increase in the MCPA content when alkaline hydrolysis was included during the sample preparation.

Løkke proposed a procedure for the analysis of 2,4-D and dichlorprop in cereals by the inclusion of a chemical hydrolysis step followed by an enzymatic deconjugation step [9]. This sample preparation procedure was compared with other two approaches not entailing any hydrolytic steps. The final results shown approx. ten and five times higher detection of dichlorprop and 2,4-D, respectively, when the hydrolytic process was included during the sample preparation.

An extensive research was carried out by Chkanikov et al. [10]. The main objectives were the investigation of the different metabolic pathways of 2,4-D in several plants (including cereals) and the use of an acidic protocol for the hydrolysis of the metabolites and the consequent release of the free 2,4-D. The concentration of 2,4-D increased approx. three times when hydrolysis was performed.

Two different extraction procedures including a hydrolysis step for the determination of mecoprop residues in barely were compared by Cessna [11]. In one of the two procedures, the alkaline deconjugation was carried out before the extraction and in the second one after the extraction. The latter protocol showed better repeatability results. Nevertheless, there was no increase in the free acid signal reported by the author.

In 2007, a standardized method for the analysis of acidic pesticides in wheat flower, including the option for alkaline hydrolysis, was delivered to the participants of the European Proficiency Test for Single Residue Method (EUPT-SRM2) [12]. As reported in [4], the wheat test material was cultivated applying MCPA in the field, thus containing the relative conjugated residue. The same approach was followed in 2009 for the EUPT-SRM4. Oat entailing incurred residues of dicamba was delivered to the participants. Based on the final results, both EUPTs showed a tangible raise of MCPA (7.1-fold increase) and dicamba (2.5-fold increase) concentrations after the alkaline hydrolysis.

In 2017, an analytical approach for the determination of acidic pesticides together with their esters and conjugates was reported [13]. This procedure is based on an alkaline hydrolysis step (30 min at 40 °C) followed by the Quick Easy Cheap Effective Rugged Safe (QuEChERS) extraction, for the residue determination of 2,4-D, dichlorprop, fluazifop, haloxyfop, MCPA, and 4-(4-chloro-2-methylphenoxy)butanoic acid (MCPB). Due to the unavailability of acidic pesticides’ conjugates as standards, their method development was carried out by the use of esters for the evaluation of the hydrolysis step. Nevertheless, the method was applied in twenty food samples characterized by the presence of incurred residues of acidic pesticides. The application of an alkaline hydrolysis step always showed an increase in the final concentration of the respective free compounds. Additionally, the method was tested in six different German laboratories (not involved during the method development) and a residue amount up to six times higher was detected if the hydrolysis step was included in the analytical procedure.

Recently, analytical standards for several phenoxyacid glucoside conjugates have become available. In the present research, an analytical method for the intact content of acidic herbicides’ glucosides and a second method for the analysis of their respective free acids after enzymatic deconjugation were developed and validated. The latter one can be considered as a selective manner to obtain the deconjugation of glucoside metabolites. Moreover, the enzymatic deconjugation is milder compared to the harsh chemical deconjugation methods which may result in degradation of certain parent pesticides. In the analytical observational report from the EU Reference Laboratory for Pesticides Requiring Single Residue Methods, poor recoveries of the free acid fenoxaprop were reported after the alkaline hydrolysis of its ester [4]. The poor stability of this pesticide under alkaline conditions was also confirmed conducting the hydrolysis directly to a mixture of free acids.

In this work, various enzymes and conditions for deconjugation were investigated to achieve quantitative conversion into the free acids. For the optimum enzyme/conditions, the method was validated for cereals (wheat), oilseeds (linseed), and a baby food product (rice-based). In addition, a QuEChERS-based method for direct determination of the available four glucoside conjugates was also validated for cereals and oilseeds. Finally, the suitability of the enzymatic deconjugation strategy was tested in fifteen samples with incurred residues, including a comparison with the acetate-buffered QuEChERS extraction technique with and without alkaline hydrolysis.

## Materials and methods

### Chemicals, standards, enzymes, and samples

#### Chemicals

Acetonitrile (ACN) was purchased from Biosolve (Valkenswaard, The Netherlands). Acetic acid (AA) (99.0%), ammonium sulphate (≥ 99.0%), sulfuric acid (99.0%), sodium acetate anhydrous, magnesium sulphate, and sodium hydroxide (≥ 98.0%) were purchased from Sigma Aldrich Chemie B.V. (Zwijndrecht, The Netherlands). Formic acid (≥ 99.0%) was obtained from VWR (Lutterworth, UK). Water was obtained from a Milli-Q water purification system from Millipore (Burlington, MA, USA).

#### Standards

High purity (> 99%) free acid pesticide standards of dichlorprop, haloxyfop, and MCPA were purchased from LGC-Dr. Ehrenstorfer (Augsburg, Germany), and 2,4-D from Honeywell Riedel-de Haën AG (Seelze, Germany). The corresponding glucosides, namely dichlorprop glucoside (95.6%), haloxyfop glucoside (95.8%), MCPA-glucoside (94.5%), and 2,4-D glucoside (85.4%), were obtained from HPC Standards GmbH (Borsdorf, Germany). Isotope-labeled internal standards (ILISs) of dichlorprop-d6 (98.4%) and 2,4-D-d3 (97%) were purchased from LGC-Dr. Ehrenstorfer, and haloxyfop-d4 (98.0%) and MCPA-methyl-d3 (98%) from TRC (North York, Canada). Stock solutions of the free acid pesticides and their respective ILISs were prepared in methanol at a concentration of 1 mg mL^−1^. Glucosides stock solutions were prepared in ACN at a concentration of 1 mg mL^−1^ for MCPA-glucoside and at 0.5 mg mL^−1^ for the other three metabolites. All the standard solutions were stored at − 20 °C.

#### Enzymes

Two enzymes, namely β-glucosidase (*Almond*) and α- and β-glucosidase (*Aspergillus niger*), were obtained from Sigma Aldrich Chemie B.V. The other six enzymes (α-glucosidase (*Yeast*), α-glucosidase (*Aspergillus niger*), β-glucosidase (*Aspergillus niger*), β-glucosidase (*Thermotoga maritima*), and β-glucosidase (*Phanerochaete chrysosporium*)) were purchased from Megazyme (Bray, Ireland), and β-glucosidase (*Bacteroides fragilis*) from Prozomix Limited (Haltwhistle, UK). All enzymes tested can be considered easy to handle. More features concerning enzymes are reported in Table S1.

#### Samples

Wheat flour, linseed, and rice-based infant formula samples were purchased in a local organic shop and were employed for validation purpose. Wheat flour and rice-based infant formula were not subjected to any pre-treatment. The linseed sample was homogenized by a conventional milling procedure at ambient temperature. Samples were stored at − 20 °C until use.

The following fifteen samples, with incurred residues, were analyzed: hempseeds, linseeds, maize, millet, peas, rapeseed meal (× 2), rapeseed cake, sunflower seed meal (× 2), grapefruit (× 3), lemon, and mandarin.

### Sample preparation

Sample preparation was based on acetate-buffered QuEChERS [14] with some modifications reported below. In general, 2.50 ± 0.05 g of sample was weighted in a 50-mL centrifuge tube, 7.5 mL of water was added, and 30 s of vortex mix was carried out. For the recovery studies, samples were fortified at this stage. Afterwards, 10 mL of ACN (1% AA) extraction solvent was added and the tubes were agitated in an automatic axial extractor (Agytax®) during 3 min. Next, a salt mixture of magnesium sulphate (4 g) and sodium acetate (1 g) was added, and the tube was immediately shaken manually and then for 1 min in the agytax machine, to induce phase separation. The tubes were then centrifuged at 4000 r.p.m. for 5 min at 10 °C. Finally, 500 µL of extract was pipetted into the mini-uniprep PTFE filer vial (45 µm), whereupon they were ready to be injected in the UHPLC-MS/MS system.

#### Intact glucosides: direct analysis

For the analysis of intact glucoside content in wheat, the procedure reported above was used with one modification: the volume of ACN (1% AA) and the 7.5 mL of water were added at the same time. This decision was initially based on low recoveries % obtained for MCPA-glucoside (< 40%) in the early stages of method development. The presence of intrinsic enzymes capable to partially hydrolyze glucosides (undesirable within this approach) was supposed and then confirmed. The prompt addition of the acidified acetonitrile leads to the denaturation of proteins, causing the loss of enzymes’ biological activity (“Method development” section).

#### Intact glucosides: indirect analysis (after enzymatic conversion into the free acid)

**Fig. 1:**
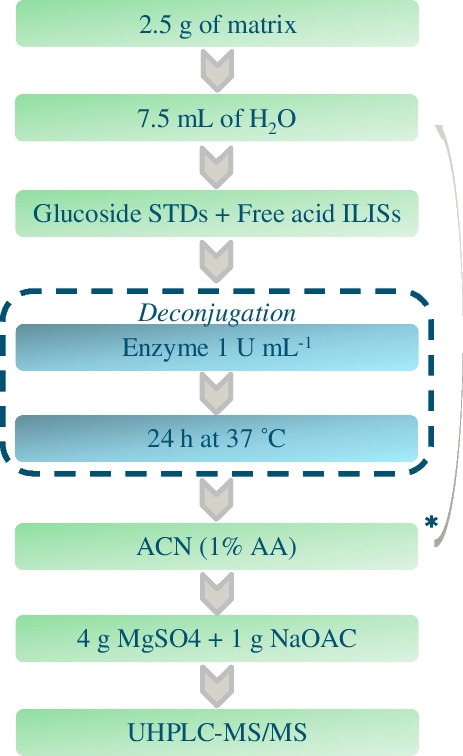
Combined sample preparation scheme for (in)direct analysis of phenoxy acid glycosides. *For the evaluation of intact glucosides in wheat, ACN (1% AA) has been added together with the water in order to inactivate intrinsic enzyme activity, and the deconjugation step is omitted

Regarding the sample preparation procedure involving the enzymatic deconjugation, 7.5 mL of acetate-buffered water (0.25 M, pH 4.0) containing 1 U mL^−1^ of enzyme was added to the 2.50 g of sample, together with the glucosides’ standards and the free acids’ ILISs. Subsequently, 24-h deconjugation at 37 °C in a water bath was accomplished. At the end of the incubation period, 10 mL of ACN (1% AA) was added and all the following extraction steps were carried out as reported above. Figure 1 illustrates a simplification scheme of the sample preparation.

#### Alkaline hydrolysis of samples with incurred residues

Deconjugation by alkaline hydrolysis was based on [4]. For this, 2.50 ± 0.05 g of sample was weighed in a 50-mL centrifuge tube; then, 7.5 mL of water was added together with the free acids’ ILISs, followed by 30 s of vortex mix. Afterwards, 10 mL of ACN (1% AA) and 2 mL of NaOH (5N) were added and the tubes were agitated in the agytax machine during 3 min. The tubes were placed in a water bath for 120 min at 40 °C. After the cooling down of the tubes, 2 mL of H_2_SO_4_ (5N) was added and the tube was shaken vigorously. Finally, all the following extraction steps were carried out as reported above.

### Instrumental conditions

The analyses were carried out by the use of a Nexera X2 LC-system (Shimadzu, Kyoto, Japan). The system was equipped with two LC-30AD pumps, a DGU-20A 5R degassing unit, a CTO-20AC oven, and a SIL-30AC autosampler. The UHPLC system was coupled to a hybrid quadrupole/linear ion trap mass spectrometer 6500^+^ QTRAP (Sciex Instruments, Concord, Ontario, Canada). An electrospray ion source (ESI) was employed for the ionization purpose. An acquity UPLC BEH C18 column (130 Å, 1.7 µm, 2.1 mm × 100 mm) from Waters Corp. (Milford, MA, USA) was employed as separation column and it was maintained at a constant temperature of 35 °C. Water with 0.1% of formic acid and ACN with 0.1% of formic acid were elution solvents A and B, respectively. The chromatographic run was carried out by applying the following gradient: 0–7 min, 10–90% B, hold for 2 min, 9–9.3 min, 90–10% B. The injection volume, the flow rate, and the injector temperature were 2 μL, 0.3 mL min^−1^, and 15 °C, respectively. The total run time was 12 min, including the re-equilibration period.

The QTRAP-MS system was employed in the multiple reaction monitoring mode (MRM) applying unit mass resolution for both Q1 and Q3. The ESI source was operated in negative ionization mode.

The following parameters were applied to the MS system: ion spray voltage: − 4500 V; curtain gas flow: 30 L min^−1^; interface temperature: 400 °C; ion spray gasses were maintained at a pressure value of 40 and 70, respectively. For quantitative purposes, two selected reaction monitoring transitions for each compound were acquired by the application of a dwell time of 20 ms. Formic acid adducts were monitored for all glucoside compounds. All the MS/MS transitions together with the relevant parameters (declustering potential, entrance potential, collision energy, and cell exit potential) are reported in Table S2. Data were processed through the use of SCIEX OS software (v. 2.2).

### Methods validation parameters

The validation of the methods was performed according to the SANTE/11312/2021 guideline [15]. Briefly, the linearity of each analyte was assessed by performing six-point calibration (for details, see the supplementary material). The deviation of back-calculated concentration from the true concentration should be ≤ 20%. The matrix effect was assessed by comparing the response of the standards in matrix with the one of standards prepared in solvent. Recovery experiments were performed fortifying the matrices at different levels (*n* = 6 for each level). The average recoveries should be between 70 and 120%.

The precision (or repeatability) of the method was assessed by calculating the relative standard deviation (RSD%) of each analyte in the three matrices spiked at the levels reported in the respective tables. According to SANTE/11312/2021, the RSD% of each level spiked should be ≤ 20%. The limit of quantification (LOQ) was defined as the lowest concentration of the analyte that has been validated with acceptable trueness (recovery) and precision (RSD %) by applying the complete analytical method and identification criteria (± 0.1 min of retention time shift and ion ratio within ± 30% of average of calibration standards from the same analytical sequence).

## Results and discussion

### Direct analysis of intact glucosides in wheat and linseed

#### Method development

**Fig. 2:**
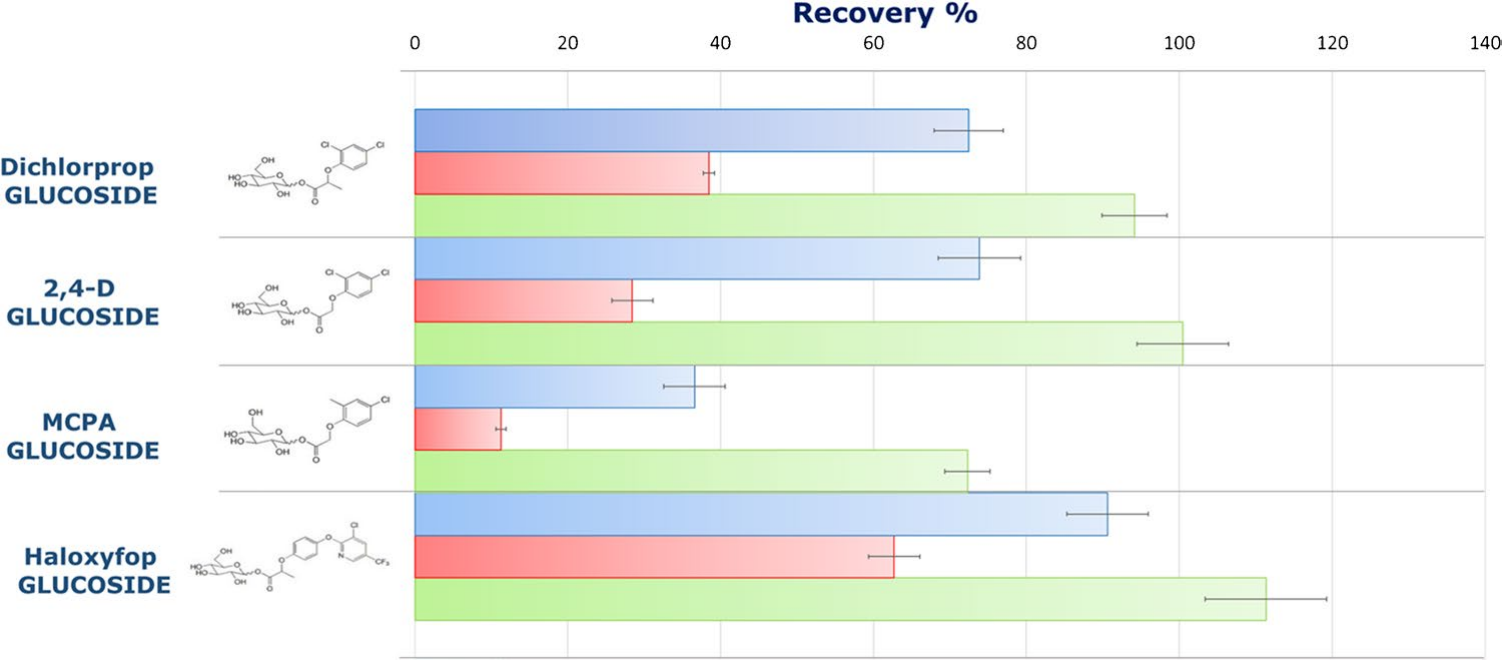
Confirmatory experiment for the evaluation of intrinsic enzymatic activity in wheat. Recoveries % are relative to the average values obtained at the three fortification levels. Blue bars = addition of water (5 min soak) followed by addition of acidic ACN (conventional procedure); red bars = addition of water (30 min soak) followed by addition of acidic ACN; green bars = addition of water in conjunction with acidic ACN

During the method development, the intact glucosides recoveries at three fortification levels (*n* = 18) in wheat appeared to be good for haloxyfop glucoside (avr. 90%), fairly good for 2,4-D glucoside and dichlorprop glucoside (avr. 74% and avr. 72%, respectively), but unacceptable for MCPA-glucoside (avr. 36%). Nevertheless, the RSD% was always < 20%. Besides, satisfactory recoveries percentages were obtained for all the four glucosides in linseed (ranging between 76 and 103%). An hypothesis was firstly assumed and then confirmed, focused on the presence, in wheat sample, of intrinsic enzymes capable to partially hydrolyze glucosides after water was added to the wheat flour. For the determination of the intact glucosides, hydrolysis should be prevented, and the intrinsic enzymes needed to be deactivated. To this end, the addition of acidic ACN was done together with the water, rather than first soaking with water and then adding the acetonitrile for extraction. The effectiveness of this approach was confirmed by comparing three slightly different sample preparation approaches, herein briefly described: (I) addition of water (5 min soak) followed by addition of acidic ACN; (II) addition of water (30 min soak) followed by addition of acidic ACN; (III) addition of water in conjunction with acidic ACN. The confirmatory experiment outcomes are illustrated in Fig. 2. The blue bars show the average recoveries (%) by the use of the conventional QuEChERS procedure (I): initial homogenization of the sample with water, followed by the analytes fortification, and the addition of the organic solvent. As described at the beginning of the paragraph, MCPA-glucoside was the compound with the lower recovery. The red bars showed the results if a longer time (30 min) was used for soaking after the fortification with glucosides (to the aqueous solution) and before the addition of the organic solvent (II). A severe reduction in the recovery % for all the compounds is evident. The average recovery for MCPA-glucoside decreased to 11%, and up to 28, 39, and 63% for 2,4-D glucoside, dichlorprop glucoside, and haloxyfop glucoside, respectively. If water and acidic ACN were added at the same time (III) and before the metabolite standards (green bars), all the recovery values improved to approx. 70% for MCPA-glucoside and > 90% for the other three compounds.

MCPA-glucoside was found to be the compound most susceptible to the enzymatic activity. Of the four glucosides tested, the phenoxyacid glucosides were more prone to the deconjugation by wheat enzymes than the aryloxyphenoxyacid-glucoside (haloxyfop glucoside). This might be caused by the presence of an additional aromatic ring (the pyridine substituent) inside the haloxyfop glucoside structure, which can probably play a crucial role in the bonding interaction between the active sites of both enzyme and metabolite.

#### Method validation

The validation was carried out for both wheat and linseed matrices. To compensate for matrix effects, quantitation was performed by the use of matrix-matched calibration line in a range of 1.25–25 ng mL^−1^ (6 calibration levels) in triplicate. The linearity results are reported in Table S3. The deviation of the back-calculated concentration percentage (BCC%) was generally within ± 10%.

Performance criteria for the four glucoside were assessed at 10, 20, and 50 µg kg^−1^ (six replicates each) in both matrices (Table 1). The recovery results were similar in both matrices. The lower recovery values were 67% and 68% at a level of 50 µg kg^−1^ for MCPA-glucoside in wheat and linseed, respectively. The RSD% was ≤ 12% and ≤ 10% for wheat and linseed, respectively.

**Table 1:** Spiked levels, extraction recoveries %, and relative standard deviation % (RSD%) obtained for the analysis of intact glucosides in wheat and linseed

Compound	Spike level µg kg^−1^	Wheat		Linseed	
		Recovery %	RSD %	Recovery %	RSD %
2,4-D glucoside	10	107	10	109	10
	20	95	10	102	6
	50	99	9	99	5
Dichlorprop glucoside	10	99	7	102	5
	20	91	6	87	8
	50	92	3	77	4
MCPA glucoside	10	79	6	88	4
	20	70	5	72	8
	50	67	2	68	3
Haloxyfop glucoside	10	115	12	104	4
	20	113	4	91	3
	50	106	3	89	3

Matrix effects were calculated by comparing the response of the analytes in matrix and in solvent at the same concentration level (25 ng mL^−1^). Detailed data are provided in Table S4. Significant suppression (*T*-test, *α* = 0.05) was observed in most cases, between − 18 and − 25% for wheat, and between − 22 and − 31% for linseed. Ion ratios were within ± 30% of the average value of calibration standards.

For all the four glucosides and for both matrices, an LOQ of 10 µg kg^−1^ was set, according to the SANTE/11312/2021 guideline.

### Indirect analysis of glucosides by enzymatic conversion into the free acid

#### Method development

The aim of this analytical approach was to optimize a procedure for the full and selective deconjugation of the metabolites followed by QuEChERS extraction, and to quantify the respective free acid by UHPLC-MS/MS. In order to properly quantify the free acid content starting from the metabolites, the differences in molecular weights were taken into account.

**Fig. 3:**
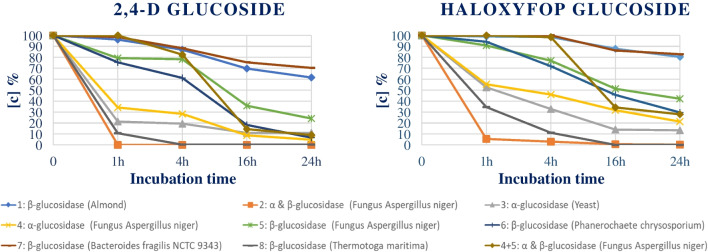
Evaluation of the enzymatic activity. The number preceding the enzyme name refers to Table S1

The glucosidase activity of several enzymes was investigated. Eight enzymes and a mixture of two out of the eight have been tested to evaluate their activity towards the glucosides of acidic herbicides. Preliminary tests were performed in acetate-buffered water solution (0.23 M) of a mixture of 2,4-D glucoside and haloxyfop glucoside at an initial concentration of 25 ng mL^−1^. The enzyme concentration was arbitrarily set at 1 U mL^−1^, following the supplier recommendations for the optimum temperature and pH (Table S1). The incubation time was set at 1, 4, 16, and 24 h. Figure 3 illustrates the decrease in [c]% at different time intervals obtained by the use of nine different enzymes for the 2,4-D and haloxyfop glucosides. The decrease of the glucosides concentration and the increase of the respective free acidic herbicide (data herein not reported) were monitored. After 24 h of incubation, most of the enzymes tested did not lead to quantitative deconjugation. Two enzymes, α- and β-glucosidase (fungus *Aspergillus niger*) and β-glucosidase (*Thermotoga maritima*), showed promising results in standard solutions, leading to a full conversion of the two glucosides into their respective native herbicides after 16 h of incubation. The enzyme α- and β-glucosidase from fungus *Aspergillus niger* proved to be more practical, with an incubation temperature of 37 °C compared to the 90 °C for the β-glucosidase from *Thermotoga maritima*. Therefore, the enzyme from fungus *Aspergillus niger* was selected for testing the deconjugation in presence of matrix and for the further validation experiments.

The presence of matrix and the concentration of the enzyme on the deconjugation were also evaluated (Table S5). The experiment was carried out by fortifying each glucoside at 20 ng mL^−1^ in water solution and three different commodities, namely wheat, linseed, and dry peas, as an additional matrix in which acid herbicides have been found in our laboratory. These samples were incubated for 24 h at 37 °C together with 0.1 U mL^−1^ and 1 U mL^−1^ of enzyme. Afterwards, acetate-buffered QuEChERS extraction was performed before sample analysis. The % decrease of each glucoside was monitored. As reported in Table S5, the use of 10 times lower enzyme [c] resulted in an insufficient activity for the full conversion of all the glucosides. The presence of matrix revealed an increase in the deconjugation efficiency compared to water for all the glucosides in the three matrices, except for haloxyfop glucoside in wheat. Furthermore, the use of 1 U mL^−1^ is necessary to obtain the full conversion of glucosides into the respective free acidic compounds. Only in wheat the [c] % of dichlorprop glucoside and haloxyfop glucoside, using 1 U mL^−1^ of enzyme at 24 h of incubation, resulted to be 10% and 3.4% respectively.

Based on these results, the α- and β-glucosidase from fungus *Aspergillus niger* (enzyme n˚2) at a concentration of 1 U mL^−1^ was selected for the validation of the method. The enzymatic deconjugation reaction was conducted at 37 °C for 24 h.

#### Method validation (wheat and linseed)

The developed method was validated for wheat and linseed by spiking with the acidic herbicides’ glucosides and quantitative determination of the generated free acidic compounds. The calibration line was prepared in solvent in a range from 0.25 up to 20 ng mL^−1^ (six calibration levels) in triplicate. The ILISs of the four free acidic herbicides were added (at a concentration of 10 ng mL^−1^) to the matrix before the deconjugation step to compensate for matrix effect, and for any losses during the incubation and extraction. Hence, the recoveries as determined here are apparent recoveries [15], rather than recoveries as defined in SANTE/11312/2021 [16]. The linearity results obtained for both matrices are reported in Table S6. The BCC% was ≤  ± 10% in all cases. The RSD% of the triplicate injections of calibrants was ≤ 11%, except for 2,4-D (25% at 0.25 ng mL^−1^).

The trueness of the method has been assessed at five levels in the range 2.5 to 38 µg kg^−1^ (*n* = 6) and the results are reported in Table 2. All the recovery results were between 70 and 120%. The lowest recovery was obtained for dichlorprop (76%) at 7 and 14 µg kg^−1^, while values above 100% were observed for haloxyfop (max 117% at 19 µg kg^−1^) in linseed matrix. All the recovery values calculated in wheat matrix were in a range between 89 and 98%. The total average recovery was 92% and 97% in wheat and linseed, respectively. The RSD% was ≤ 13% (avr.7%) and ≤ 9% (avr.5%) for wheat and linseed, respectively. Matrix effects were not assessed, as correction was made by the use of ILISs. Ion ratios were within ± 30% of the average value of calibration standards.

**Table 2:** Spiked levels of glucoside conjugates, expressed as free acids, apparent recoveries %, and relative standard deviation % (RSD%) obtained for the analysis of free acidic herbicides after the deconjugation of their respective glucosides in wheat and linseed

Compound	Spike level µg kg^−1^	Wheat		Linseeds	
		Recovery %	RSD %	Recovery %	RSD %
2,4-D glucoside	2.5	92	13	103	7
	5	96	12	105	7
	7	91	10	91	4
	12	92	4	99	3
	27	89	3	87	3
Dichlorprop glucoside	2.5	92	10	104	9
	5	92	13	98	6
	7	95	9	76	4
	14	92	3	76	5
	28	92	4	78	4
MCPA glucoside	2.5	90	4	99	8
	5	92	13	99	6
	6	89	4	90	3
	12	91	3	91	3
	25	90	2	90	2
Haloxyfop glucoside	2.5	98	8	105	3
	5	97	8	106	4
	10	92	3	112	3
	19	91	2	117	7
	38	90	3	113	5

For all the four acidic herbicides’ conjugates, an LOQ of 2.5 µg kg^−1^ was applicable in both matrices, according to the SANTE/11312/2021 guideline.

#### Method validation (rice-based infant formula)

The enzymatic deconjugation approach, developed and validated for cereal-based matrices, was also applied to a rice-based infant formula. In accordance with Article 4 of Regulation (EU) 2016/127, the total residue of haloxyfop in infant formulas should not exceed a concentration of 3 µg kg^−1^ [17Due to the low concentration requested by the Regulation reported above, the validation of a method for the intact glucoside content in rice-based infant formula was not included in the validation plan, inasmuch the LOQ obtained for wheat and linseeds (10 µg kg^−1^) exceed the MRL set for haloxyfop. The sample preparation and the analytical approach were the same as reported in the “Method validation (wheat and linseed)” section, but lower calibration and fortification levels were tested. The calibration line was prepared in solvent in a range from 0.1 up to 5 ng mL^−1^ (six calibration levels) in triplicates. The ILISs of the four free acidic herbicides were added at a concentration of 2 ng mL^−1^ to compensate for matrix effect, and for any potential sample preparation losses. In Table S7 are shown the linearity results for the free acidic herbicides. At the lowest calibration level (0.1 ng mL^−1^), 2,4-D was not detected. The BCC% ranging between − 19% (MCPA at 0.1 ng mL^−1^) and + 15% (MCPA at 0.5 ng mL^−1^). The RSD% was always ≤ 15%, with an average value of 5%.

**Fig. 4:**
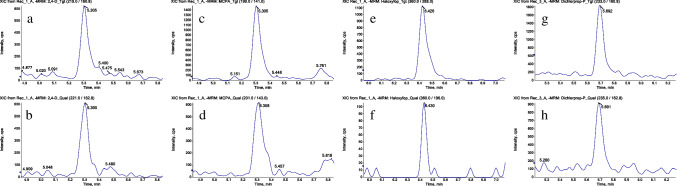
Extracted ion chromatograms for the quant/qual peaks of 2,4-D (**a**/**b**), MCPA (**c**/**d**), haloxyfop (**e**/**f**) at 1 µg kg^**−**1^, and dichlorprop (**g**/**h**) at 3 µg kg^**−**1^ relative to the rice-based infant formula

In order to fulfill the requirements of the EU Regulation, the method validation criteria were assessed fortifying the rice-based infant formula at two very low concentrations: 1 and 3 µg kg^−1^. In Table 3 are reported the trueness results based on six replicates for each level. The recoveries at 3 µg kg^−1^ ranging between 50% (dichlorprop) and 84% (2,4-D), while the RSD% was always ≤ 10%. At the lowest fortification level, dichlorporp was not detected, MCPA and 2,4-D recoveries were ≥ 70%, and haloxyfop recovery was slightly below 70%. Also in this case matrix effect was not assessed, because of the use of ILISs. The extracted ion chromatograms of the free acidic herbicides’ signals at the lowest achievable fortified levels are shown in Fig. 4. The peaks of 2,4-D (Fig. 4a and b), MCPA (Fig. 4c and d), and haloxyfop (Fig. 4e and f) are relative to the 1 µg kg^−1^ fortification level, while the peak of dichlorprop (Fig. 4g and h) is relative to the 3 µg kg^−1^ fortification level. The ion ratios were within the ± 30% of the average of calibration standards with the exeption of haloxyfop at the 1 µg kg^−1^ level, where the signal for the qualifier was really close to the detection limit.

**Table 3:** Spiked levels of glucoside conjugates, expressed as free acids, apparent recoveries %, and relative standard deviation % (RSD%) obtained for the analysis of free acidic herbicides after the deconjugation of their respective glucosides in the rice-based infant formula

Compound	Spike level µg kg^−1^	Rice-based infant formula	
		Recovery %	RSD %
2,4-D glucoside	1	76	12
	3	84	4
Dichlorprop glucoside	1	N/A	N/A
	3	50	10
MCPA glucoside	1	70	10
	3	76	10
Haloxyfop glucoside	1	69	8
	3	67	8

The LOQ values were 3 µg kg^−1^ for dichlorprop and 1 µg kg^−1^ for 2,4-D, MCPA, and haloxyfop. Consequently, the method herein proposed is suitable for the determination of concentrations ≥ 1 µg kg^−1^ of the sum of free and glucoside of haloxyfop content in rice-based infant formulas.

### Analysis of real samples with incurred residues

Fifteen matrices with incurred residues were subjected to analysis in order to test the applicability of the proposed enzymatic deconjugation strategy, and to compare the method to (a) a method without deconjugation, and (b) an existing method that uses alkaline hydrolysis. The analyzed matrices belong to three different commodity groups according to SANTE/11312/2021 guideline, namely high oil content and very low water content, high starch and/or protein content and low water and fat content, and high acid content and high water content. Two out of the four free acidic herbicides were detected in the real samples, namely 2,4-D and haloxyfop. Three samples were characterized by the presence of both pesticides, while exclusive 2,4-D was detected in twelve matrices. The analysis of incurred samples was carried out applying three different approaches: acetate-buffered QuEChERS, acetate-buffered QuEChERS including alkaline hydrolysis (considered to convert “any” conjugate and esters into free acids), and acetate-buffered QuEChERS after the enzymatic deconjugation of glucosides developed and validated in this work. The comparison of the three different approaches helps to confirm the applicability of the enzymatic method compared to the two most common used methods of analysis.

The results are summarized in Table 4.

**Table 4:** Results relative to the quantification of 2,4-D and haloxyfop in samples with incurred residues extracted by the three different strategies

N˚	Commodity	Pesticide	QuEChERS (Q) [c] µg kg^−1^	Enzymatic (E) [c] µg kg^−1^	Alkaline (A) [c] µg kg^−1^	Ratio E/Q	Ratio A/Q	Ratio E/A
1	Hempseed	2,4-D	17.0	27.2	25.4	160%	150%	107%
2	Linseeds	2,4-D	10.7	12.7	17.3	119%	162%	73%
2	Linseeds	Haloxyfop	24.3	36.2	35.4	149%	146%	102%
3	Maize	2,4-D	8.39	8.4	10.1	100%	121%	83%
4	Millet	2,4-D	22.9	24.3	22.5	106%	99%	108%
5	Peas	2,4-D	28.5	37.8	37.5	132%	132%	101%
6	Rapeseed meal S1	Haloxyfop	22.7	27.9	31.5	123%	138%	89%
7	Rapeseed meal S2	Haloxyfop	42.6	51.7	59.5	121%	140%	87%
8	Rapeseed cake	Haloxyfop	132	188	205	143%	155%	92%
9	Sunflower seed meal S1	2,4-D	19.1	20.4	23.0	107%	120%	89%
9	Sunflower seed meal S1	Haloxyfop	3.7	3.8	6.6	101%	176%	57%
10	Sunflower seed meal S2	2,4-D	14.7	15.8	17.6	108%	120%	90%
10	Sunflower seed meal S2	Haloxyfop	12.1	12.6	20.8	104%	172%	60%
11	Grapefruit S1	2,4-D	49.8	102	104	205%	210%	98%
12	Grapefruit S2	2,4-D	86.7	232	234	267%	270%	99%
13	Grapefruit S3	2,4-D	106	277	263	263%	249%	105%
14	Lemon	2,4-D	37.4	70.4	94.8	188%	254%	74%
15	Mandarin	2,4-D	126	234	310	185%	245%	76%

In most of the cases, the inclusion of the deconjugation/hydrolysis steps led to an increase of the concentration of the total free acid content. For haloxyfop in oil seeds, this was a factor 1.4–1.7. For 2,4-D, this ranged from no increase to a factor 1.6 in dry matrices (oil seed, cereals, peas), and a factor 2.1–2.7 in citrus. The differences in total free acids between the two deconjugation methods were minor for 13 of the 15 samples. The exceptions concerned haloxyfop in sunflower seed meals S1 and S2: no increase with the enzymatic method, 1.7-fold increase for the alkaline hydrolysis. This could indicate that for this particular pesticide/matrix combination other conjugates and/or esters may have been present in the sample material. The highest concentration’s increase was observed for 2,4-D in grapefruit S2 (sample n˚12) for which a 2.7-fold increase was found if enzymatic or alkaline hydrolysis was included in the sample preparation.

**Fig. 5:**
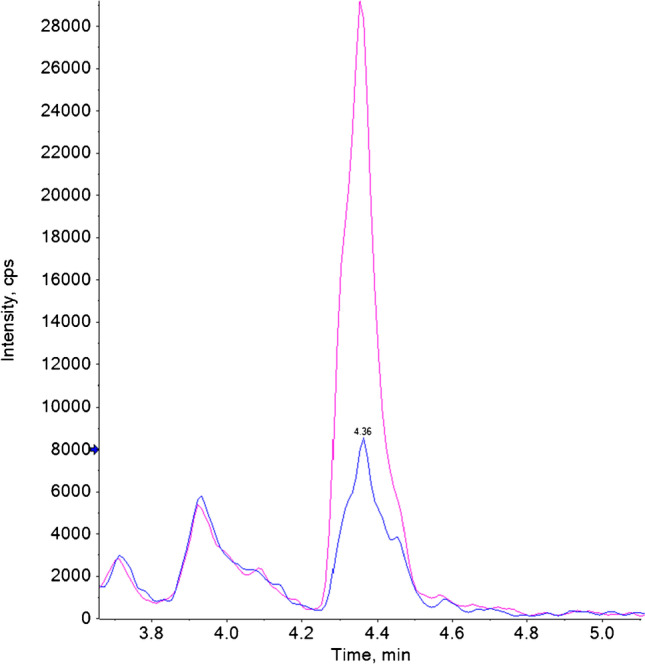
Extracted ion chromatogram (EIC) for 2,4-D glucoside in mandarin sample using conventional QuEChERS extraction without deconjugation. Extract of the sample without (blue) and with (pink) addition of the standard of 2,4-D glucoside

Noteworthy is the detection of the 2,4-D glucoside’s peak in all the three grapefruit samples, in lemon and in mandarin. The peak identification was confirmed against the solvent standard and further by over spiking the extract with the analytical standard (illustrated in Fig. 5, and more details in Figure S1).

Although in citrus fruits the application of the pesticide takes place in the form of esters relatively short before harvest to reduce preharvest drop of mature fruit, our results confirm that esters in this case are less or not relevant for the total 2,4-D residue. This is in line with the reported rapid hydrolysis of esters after application [5].

## Conclusion

In the present research, a novel method for the total free acidic herbicides content, after enzymatic deconjugation, was proposed and validated in three relevant matrices. This approach can be considered reliable and specific for releasing the parent pesticide from the glucoside metabolite(s), without risking any further hydrolysis as may happen with alkaline hydrolysis. The enzyme α- and β-glucosidase (fungus *Aspergillus niger*) demonstrated satisfactory deconjugation power together with high repeatability in the presence of all the three matrices included in this study.

A method for the direct determination of the intact glucoside content was also optimized and validated. The method demonstrated good extraction recoveries and repeatability, although the few analytical standards on the market make it applicable to real samples only for a limited number of pesticide conjugates.

In both cases, method validation was successful and LOQs down to 1 µg kg^−1^ were achieved.

Based on the results obtained for the analysis of samples with incurred residues, the use of the enzymatic deconjugation is comparable to the existing alkaline hydrolysis method with the benefit of preventing hydrolysis of other functional groups present in the parent pesticides. On the other hand, the enzymatic approach resulted to be more time consuming as it requires a 24-h incubation.

Finally, future investigation will be carried out to expand the number of matrices, and to include new standards when available on the market.

### Supplementary Information

Below is the link to the electronic supplementary material.

Supplementary file1 (DOCX 164 KB)
