# Supplementary material for: Development and validation of two analytical strategies for the determination of glucosides of acidic herbicides in cereals and oilseed matrices
Source: Anal Bioanal Chem. 2023 Aug 14;416(3):651–61. doi: 10.1007/s00216-023-04898-y (PMC10766664; doi:10.1007/s00216-023-04898-y)
Supplement: Supplementary file 1 — Supplementary file1 (DOCX 164 KB) [file 216_2023_4898_MOESM1_ESM.docx]

**DEVELOPMENT AND VALIDATION OF TWO ANALYTICAL STRATEGIES FOR THE DETERMINATION OF GLUCOSIDES OF ACIDIC HERBICIDES IN CEREALS AND OILSEED MATRICES**

Ivan Aloisi*, Hans Mol

*Wageningen Food Safety Research (WFSR), part of Wageningen University & Research, Wageningen, the Netherlands*

*Corresponding author

E-mail address: ivan.aloisi@wur.nl

ORCID: Ivan Aloisi: 0000-0002-3164-3928

| **N˚** | **α-glucosidase and/or**  **β glucosidase** | **Origin** | **Optimal**  **pH/T** | **Supplier** | **Cat. number** | **Stock [c]** | **Physical state** | **Cost (€)**  **(Cost × sample)** | **Storage condition** | **Stability** |
| --- | --- | --- | --- | --- | --- | --- | --- | --- | --- | --- |
| 1 | β-glucosidase | Almond | 5.0 / 37˚C | Sigma Aldrich | G4511-1KU | 10-30 U mg^-1^ | powder | 1390 (1.39) | 2-8 ˚C | Not reported |
| 2 | α & β-glucosidase | Fungus (Aspergillus niger) | 4.0 / 37˚C | Sigma Aldrich | 49291-1G | 1398 U g^-1^ | powder | 281 (0.20) | 2-8 ˚C | Not reported |
| 3 | α-glucosidase | Yeast | 6.8 / 40˚C | Megazyme | E-MALTS | 1000 U mL^-1^ | suspension | 186 (2.09) | 2-8 ˚C | >1 year |
| 4 | α-glucosidase | Fungus (Aspergillus niger) | 4.5 / 70˚C | Megazyme | E-TRNGL | 1000 U mL^-1^ | suspension | * | 2-8 ˚C | >4 year at 4 ˚C |
| 5 | β-glucosidase | Fungus (Aspergillus niger) | 4.0 / 70˚C | Megazyme | E-BGLUC | 40 U mL^-1^ | suspension | 270 (0.74) | 2-8 ˚C | >1 year |
| 6 | β-glucosidase | Phanerochaete chrysosporium | 5.0 / 70˚C | Megazyme | E-BGOSPC | 460 U mL^-1^ | suspension | * | 2-8 ˚C | >4 year at 4 ˚C |
| 7 | β-glucosidase | Bacteroides fragilis | 4.4 / 40˚C | Prozomix | PRO-E0105 | 98 U mL^-1^ | suspension | 450 (0.98) | 4 ˚C | Not reported |
| 8 | β-glucosidase | Thermotoga maritima | 7.0 / 90˚C | Megazyme | E-BGOSTM | 460 U mL^-1^ | suspension | * | 2-8 ˚C | >4 year at 4 ˚C |

**Table S1.** Detailed enzymes information reported by the suppliers.

* This product has been discontinued

**Table S2.** Tandem mass spectrometry settings for all the compound. Tgt (quantifier), Qual (qualifier), IS (internal standard), Q1 (precursor ion), Q3 (product ion), DP (declustering potential), EP (entrance potential), CE (collision energy), CXP (Collision Cell Exit Potential).

| **Compound** | **Precursor**  **ion type** | **Q1  (*m/z*)** | **Q3  (*m/z*)** | **DP  (Volts)** | **EP  (Volts)** | **CE  (Volts)** | **CXP  (Volts)** |
| --- | --- | --- | --- | --- | --- | --- | --- |
| 2,4-D_Tgt | [M-H]^-^ | 219 | 160.8 | -50 | -10 | -25 | -15 |
| 2,4-D_Qual | [M-H]^-*^ | 221 | 162.8 | -50 | -10 | -25 | -15 |
| 2,4-D-d3_IS | [M-H]^-^ | 222 | 163.8 | -50 | -10 | -25 | -15 |
| 2,4-D-glucoside_Tgt | [M+HCOO]^-^ | 427 | 161.0 | -20 | -10 | -30 | -15 |
| 2,4-D-glucoside_Qual | [M+HCOO]^-^ | 427 | 219.0 | -20 | -10 | -20 | -15 |
| Dichlorprop-P_Tgt | [M-H]^-^ | 233 | 160.8 | -50 | -10 | -20 | -15 |
| Dichlorprop-P_Qual | [M-H]^-*^ | 235 | 162.8 | -50 | -10 | -20 | -15 |
| Dichlorprop-d6_IS | [M-H]^-^ | 239 | 163.8 | -50 | -10 | -20 | -15 |
| Dichlorprop-glucoside_Tgt | [M+HCOO]^-^ | 441 | 233.0 | -40 | -10 | -20 | -15 |
| Dichlorprop-glucoside_Qual | [M+HCOO]^-^ | 441 | 161.0 | -40 | -10 | -30 | -15 |
| Haloxyfop_Tgt | [M-H]^-^ | 360 | 288.0 | -50 | -10 | -20 | -15 |
| Haloxyfop_Qual | [M-H]^-^ | 360 | 196.0 | -50 | -10 | -48 | -15 |
| Haloxyfop-d4_IS | [M-H]^-^ | 364 | 292.0 | -50 | -10 | -20 | -15 |
| Haloxyfop-glucoside_Tgt | [M+HCOO]^-^ | 568 | 360.0 | -35 | -10 | -20 | -15 |
| Haloxyfop-glucoside_Qual | [M+HCOO]^-^ | 568 | 288.0 | -35 | -10 | -30 | -15 |
| MCPA_Tgt | [M-H]^-^ | 199 | 141.0 | -50 | -10 | -16 | -15 |
| MCPA_Qual | [M-H]^-*^ | 201 | 143.0 | -50 | -10 | -16 | -15 |
| MCPA-d3_IS | [M-H]^-^ | 202 | 144.0 | -50 | -10 | -16 | -15 |
| MCPA-glucoside_Tgt | [M+HCOO]^-^ | 407 | 141.0 | -60 | -10 | -40 | -15 |
| MCPA-glucoside_Qual | [M+HCOO]^-^ | 407 | 199.0 | -60 | -10 | -20 | -15 |

*^37^Cl isotope of chlorine

**Table S3.** Linearity figure of merits relative to the quantitative analysis of intact glucosides in wheat and linseeds. The relative standard deviation (RSD%) herein reported is referred to three injections of each calibration level. The back calculated concentration (BCC%) is relative to the average % deviation of the calculated concentration (experimental) in respect to the predicted value (theoretical).

| **Compound** | **[c]_theor_  ng mL^-1^** | **Wheat** | | **Linseeds** | |
| --- | --- | --- | --- | --- | --- |
|  |  | **RSD**  **%** | **Avr. BBC %** | **RSD**  **%** | **Avr. BBC %** |
| 2,4-D glucoside | 1.25 | 13 | -1% | 7 | 13% |
|  | 2.5 | 8 | 0% | 7 | -7% |
|  | 5 | 14 | 3% | 6 | -7% |
|  | 12.5 | 8 | -5% | 7 | -3% |
|  | 20 | 5 | 4% | 6 | 5% |
|  | 25 | 10 | -2% | 7 | -1% |
| Dichlorprop glucoside | 1.25 | 14 | -2% | 2 | 21% |
|  | 2.5 | 8 | 1% | 6 | -7% |
|  | 5 | 7 | 3% | 3 | -12% |
|  | 12.5 | 8 | -4% | 7 | -10% |
|  | 20 | 4 | 4% | 4 | 7% |
|  | 25 | 9 | -2% | 1 | 2% |
| MCPA glucoside | 1.25 | 15 | -1% | 10 | 6% |
|  | 2.5 | 6 | 0% | 3 | 1% |
|  | 5 | 4 | 2% | 3 | -4% |
|  | 12.5 | 9 | -4% | 5 | -9% |
|  | 20 | 1 | 5% | 7 | 6% |
|  | 25 | 7 | -2% | 7 | 0% |
| Haloxyfop glucoside | 1.25 | 8 | -3% | 7 | 17% |
|  | 2.5 | 15 | 0% | 7 | -7% |
|  | 5 | 5 | 5% | 2 | -9% |
|  | 12.5 | 11 | -3% | 6 | -8% |
|  | 20 | 7 | 3% | 1 | 5% |
|  | 25 | 10 | -2% | 2 | 1% |

| **Compound** | **Wheat/Solvent** | **[c]_exp_ ng mL^-1^** | **ME %** | **Linseed/Solvent** | **[c]_exp_ ng mL^-1^** | **ME %** |
| --- | --- | --- | --- | --- | --- | --- |
| 2,4 D glucoside | Wheat | 21.8 | -5 | Linseeds | 26.4 | -19 |
|  |  | 26.2 |  |  | 23.0 |  |
|  |  | 25.9 |  |  | 24.6 |  |
|  | Solvent | 25.5 |  | Solvent | 28.9 |  |
|  |  | 26.1 |  |  | 30.9 |  |
|  |  | 25.9 |  |  | 32.0 |  |
| Dichlorprop glucoside | Wheat | 21.9 | **-18** | Linseeds | 25.9 | **-22** |
|  |  | 26.3 |  |  | 25.1 |  |
|  |  | 25.3 |  |  | 25.4 |  |
|  | Solvent | 29.1 |  | Solvent | 32.4 |  |
|  |  | 30.6 |  |  | 32.0 |  |
|  |  | 30.5 |  |  | 33.3 |  |
| Haloxyfop glucoside | Wheat | 21.8 | **-25** | Linseeds | 25.4 | **-23** |
|  |  | 25.4 |  |  | 25.9 |  |
|  |  | 26.3 |  |  | 24.7 |  |
|  | Solvent | 33.2 |  | Solvent | 32.2 |  |
|  |  | 31.4 |  |  | 33.0 |  |
|  |  | 33.4 |  |  | 33.0 |  |
| MCPA glucoside | Wheat | 22.4 | **-21** | Linseeds | 27.0 | **-31** |
|  |  | 25.8 |  |  | 24.2 |  |
|  |  | 25.0 |  |  | 24.0 |  |
|  | Solvent | 31.0 |  | Solvent | 37.4 |  |
|  |  | 30.5 |  |  | 36.0 |  |
|  |  | 31.7 |  |  | 36.2 |  |

**Table S4.** Matrix effect % (ME%) relative to glucosides signal comparison of wheat/solvent and linseed/solvent. In bold are reported ME% revealing significance differences via *T*-test (α=0.05).

**Table S5.** Effect of incubation time, enzyme concentration, and the presence of matrix on the deconjugation release of the free acids from the respective glucosides.

| **Time/Units mL^-1^** | **Compound** | **Water** | **Wheat** | **Linseed** | **Pea** |
| --- | --- | --- | --- | --- | --- |
|  |  | **[c] %** | **[c] %** | **[c] %** | **[c] %** |
| 0h/0U mL^-1^ | 2,4-D glucoside | 100.0 | 100.0 | 100.0 | 100.0 |
| 24h/0.1U mL^-1^ |  | 2.2 | 0.0 | 0.0 | 0.0 |
| 24h/1U mL^-1^ |  | 0.0 | 0.0 | 0.0 | 0.0 |
| 0h/0U mL^-1^ | Dichlorporp glucoside | 100.0 | 100.0 | 100.0 | 100.0 |
| 24h/0.1U mL^-1^ |  | 31.2 | 16.6 | 4.5 | 4.8 |
| 24h/1U mL^-1^ |  | 8.1 | 10.0 | 0.0 | 0.0 |
| 0h/0U mL^-1^ | Haloxyfop glucoside | 100.0 | 100.0 | 100.0 | 100.0 |
| 24h/0.1U mL^-1^ |  | 11.6 | 36.0 | 5.2 | 5.0 |
| 24h/1U mL^-1^ |  | 0.6 | 3.4 | 0.0 | 0.0 |
| 0h/0U mL^-1^ | MCPA glucoside | 100.0 | 100.0 | 100.0 | 100.0 |
| 24h/0.1U mL^-1^ |  | 9.5 | 0.0 | 0.0 | 0.0 |
| 24h/1U mL^-1^ |  | 0.0 | 0.0 | 0.0 | 0.0 |

**Table S6.** Linearity figure of merits relative to the quantitative analysis of free acidic herbicides after enzymatic deconjugation in wheat and linseeds. The relative standard deviation (RSD%) herein reported is referred to three injections of each calibration level. The back calculated concentration (BCC%) is relative to the average % deviation of the calculated concentration (experimental) in respect to the predicted value (theoretical).

| **Compound** | **[c]_theor_  ng mL^-1^** | **Wheat** | | **Linseeds** | |
| --- | --- | --- | --- | --- | --- |
|  |  | **RSD**  **%** | **Avr. BBC %** | **RSD**  **%** | **Avr. BBC %** |
| 2,4-D | 0.25 | 25 | 0% | 7 | -9% |
|  | 0.5 | 1 | -7% | 3 | 0% |
|  | 2.5 | 0 | 1% | 0 | 2% |
|  | 5 | 3 | 6% | 3 | 7% |
|  | 10 | 3 | 3% | 3 | 4% |
|  | 20 | 2 | -3% | 3 | -4% |
| Dichlorprop | 0.25 | 2 | -6% | 11 | -10% |
|  | 0.5 | 11 | 4% | 10 | 8% |
|  | 2.5 | 2 | 1% | 2 | 2% |
|  | 5 | 1 | 0% | 1 | 0% |
|  | 10 | 1 | 2% | 1 | 2% |
|  | 20 | 1 | -1% | 1 | -1% |
| MCPA | 0.25 | 4 | 2% | 3 | 3% |
|  | 0.5 | 2 | -7% | 5 | -8% |
|  | 2.5 | 1 | 3% | 1 | 4% |
|  | 5 | 2 | 1% | 2 | 1% |
|  | 10 | 2 | 1% | 2 | 1% |
|  | 20 | 0 | -1% | 0 | -1% |
| Haloxyfop | 0.25 | 10 | 6% | 10 | 0% |
|  | 0.5 | 3 | -6% | 3 | -4% |
|  | 2.5 | 1 | -2% | 1 | -1% |
|  | 5 | 2 | 2% | 2 | 3% |
|  | 10 | 1 | 3% | 1 | 4% |
|  | 20 | 3 | -1% | 2 | -3% |

**Table S7.** Linearity figure of merits relative to the quantitative analysis of acidic herbicides’glucosides after enzymatic deconjugation in rice-based infant formula. The relative standard deviation (RSD%) herein reported is referred to three injections of each calibration level. The back calculated concentration (BCC%) is relative to the average % deviation of the calculated concentration (experimental) in respect to the predicted value (theoretical).

| **Compound** | **[c]_theor_  ng mL^-1^** | **Rice-based infant formula** | |
| --- | --- | --- | --- |
|  |  | **RSD**  **%** | **Avr. BBC %** |
| 2,4-D | 0.1 | N/A | N/A |
|  | 0.25 | 5 | -11% |
|  | 0.5 | 6 | 9% |
|  | 1 | 7 | 2% |
|  | 2.5 | 2 | 1% |
|  | 5 | 7 | -1% |
| Dichlorprop | 0.1 | 7 | -7% |
|  | 0.25 | 10 | 7% |
|  | 0.5 | 6 | 0% |
|  | 1 | 9 | 0% |
|  | 2.5 | 1 | 0% |
|  | 5 | 4 | 0% |
| MCPA | 0.1 | 11 | -19% |
|  | 0.25 | 1 | 6% |
|  | 0.5 | 2 | 15% |
|  | 1 | 5 | 0% |
|  | 2.5 | 3 | -3% |
|  | 5 | 4 | 0% |
| Haloxyfop | 0.1 | 10 | -4% |
|  | 0.25 | 5 | -1% |
|  | 0.5 | 3 | 9% |
|  | 1 | 5 | -2% |
|  | 2.5 | 1 | -3% |
|  | 5 | 2 | 1% |


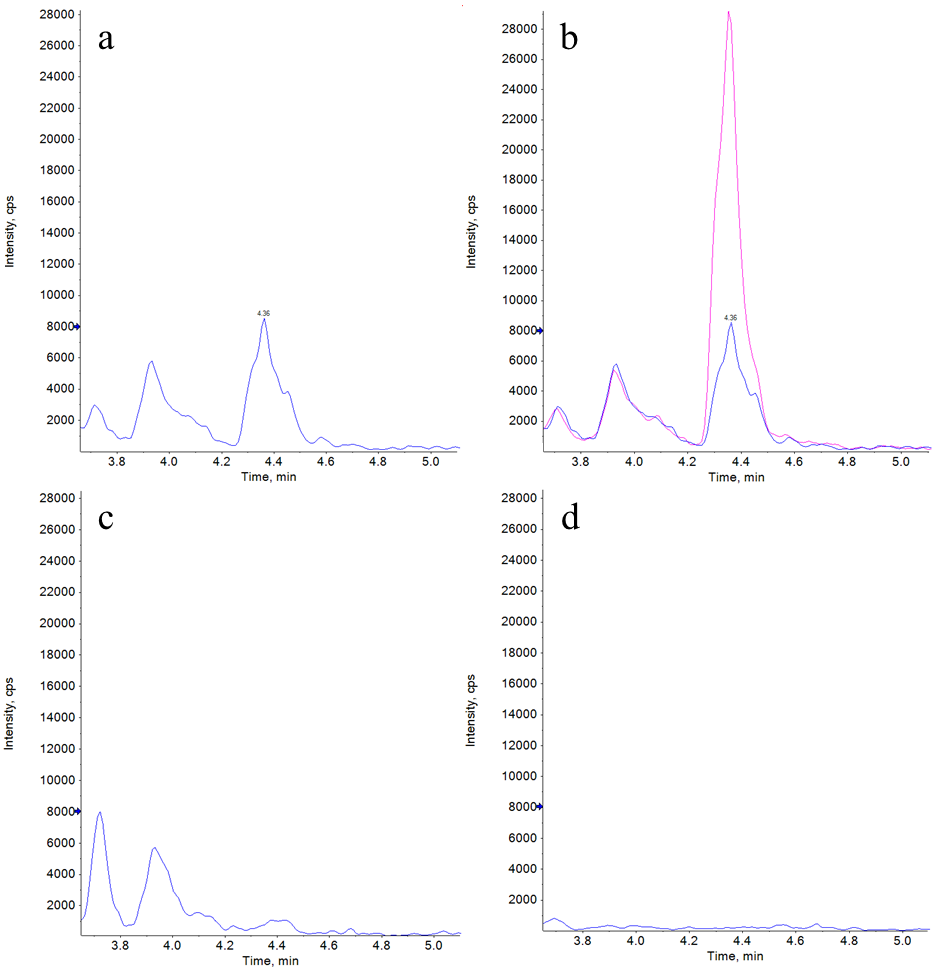


**Figure S1.** Extracted ion chromatograms (EICs) for 2,4-D glucoside in mandarin sample. Acetate buffered QuEChERS without (a) and with the addition of 2,4-D glucoside (b), after enzymatic (c) and alkaline (d) hydrolysis. In (c) and (d) the 2,4-D glucoside signal disappeared.
